# Supplementary material for: Determining the Target Population That Would Most Benefit from Screening for Hepatic Fibrosis in a Primary Care Setting
Source: Diagnostics (Basel). 2021 Sep 3;11(9):1605. doi: 10.3390/diagnostics11091605 (PMC8470742; doi:10.3390/diagnostics11091605)
Supplement: Supplementary file 1 [file diagnostics-11-01605-s001.zip › diagnostics-1311077-supplementary.pdf]

Table S1. Net benefit of FIB-4 at different Pts.

Pt	Diabetes	USG–Diagnosed NAFLD	Elevated Liver Enzyme	Metabolic Syndrome	Impaired Fasting Glucose
0.05	0.0117	0.0048	0.0014	0.005	−0.0016
0.10	0.0075	−0.008	−0.0047	−0.0023	−0.0138
0.15	0.0028	−0.0224	−0.0117	−0.0106	−0.0274
0.20	−0.0025	−0.0385	−0.0194	−0.0198	−0.0427
0.25	−0.0085	−0.0567	−0.0282	−0.0303	−0.0601
0.30	−0.0154	−0.0776	−0.0383	−0.0423	−0.0799
0.40	−0.0326	−0.1298	−0.0635	−0.0723	−0.1295
0.50	−0.0567	−0.2028	−0.0987	−0.1142	−0.1989

FIB-4, Fibrosis-4; Pt, probability threshold.
